# Arthritis-associated osteoclastogenic macrophage, AtoM, as a key player in pathological bone erosion

**DOI:** 10.1186/s41232-022-00206-w

**Published:** 2022-06-02

**Authors:** Tomoya Agemura, Tetsuo Hasegawa, Shinya Yari, Junichi Kikuta, Masaru Ishii

**Affiliations:** 1 Department of Immunology and Cell Biology, Graduate School of Medicine and Frontier Biosciences, Osaka University, 2-2 Yamada-oka, Suita, Osaka, 565-0871 Japan; 2 WPI-Immunology Frontier Research Center, Osaka University, 2-2 Yamada-oka, Suita, Osaka, 565-0871 Japan; 3 Laboratory of Bioimaging and Drug Discovery, National Institutes of Biomedical Innovation, Health and Nutrition, 7-6-8 Asagi Saito, Osaka, Ibaraki 567-0085 Japan

**Keywords:** Osteoclast, Osteoclast precursor, Macrophage, Arthritis, Two-photon microscopy

## Abstract

Osteoclasts are myeloid lineage cells with a unique bone-destroying ability that maintains bone homeostasis together with bone formation by osteoblasts. An advanced intravital imaging system using a two-photon microscopy has enabled the observation and evaluation of osteoclast dynamics and behaviors in the bone marrow of living mice. Using this system, it has become clear that pathological osteoclasts under inflamed conditions differ from physiological osteoclasts under a steady-state. Recently, we identified novel osteoclast precursors in arthritis, called arthritis-associated osteoclastogenic macrophages (AtoMs), which differentiate into pathological osteoclasts and induce inflammatory bone destruction. In this review, we introduce the in vivo imaging of physiological and pathological osteoclasts and their differentiation mechanism.

## Background

The bone is mainly formed from collagen fibers and calcium phosphates. The bone is a hard tissue, similar to tooth enamel, and plays important roles such as serving a mechanical function in the skeletons, storing reserves of mainly calcium and phosphorus, and hosting hematopoiesis in the marrow. In a steady-state, bone homeostasis (also called bone remodeling) is supported by osteoclasts and osteoblasts [1]. Osteoclasts are myeloid lineage cells that have a unique bone-destroying ability. On the other hand, osteoblasts differentiate from mesenchymal stem cells and have specialized bone-synthesizing ability. Bone remodeling involves maintaining the balance between bone resorption and bone formation. An imbalance in osteoclastic activity underlies many diseases with inflammatory bone destruction including the chronic autoimmune disease rheumatoid arthritis (RA). In Japan, there are 700,000 patients with RA. For this reason, the regulation of osteoclast functions is important for inflammatory bone destruction.

**Fig. 1:**
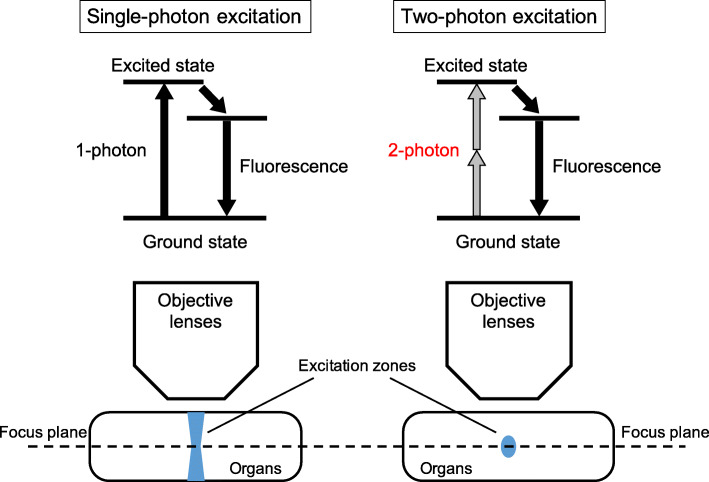
The mechanism of the two-photon microscopy. Two-photon excitation is considered extremely difficult to occur, and excitation zones occur on the focus plane only. Therefore, a two-photon microscopy can bring a high spatial resolution, low phototoxicity, and low invasiveness

Regarding the studies on osteoclasts so far, histological analyses of fixed bone sections have been used to observe osteoclasts in the bone marrow because osteoclasts bind to the bone surface via cytoskeletons such as actin and integrin. Nevertheless, histological observation cannot be used to evaluate the cell dynamics of living osteoclasts. Therefore, intravital imaging using a two-photon microscopy has become an essential tool for observing cell dynamics [2, 3]. A two-photon microscopy is a kind of fluorescence microscopy with an excitation light source that emits laser light with half the energy (twice the wavelength) of the excitation light used in a conventional fluorescence microscopy. The fluorescence is the phenomenon of energy emitted as light when the excited state of fluorescent molecules by light returns to the ground state (Fig. 1). A two-photon excitation offers the advantages of a high-spatial resolution, a comparatively long excitation wavelength that is less invasive to tissue and organs, and deep reach into the tissues and organs [4, 5]. Our laboratory was the first to visualize living cell dynamics in the bone marrow of mice by establishing an in vivo imaging system [6–9].

In this review, we introduce in vivo imaging of osteoclasts using a two-photon microscopy and the newly identified arthritis-associated osteoclastogenic macrophage (AtoM) in inflamed synovium [10].

## Main text

### Visualization of living osteoclasts in the bone marrow

To visualize osteoclasts, transgenic mice expressing fluorescent proteins under the control of the tartrate-resistant acid phosphate (TRAP) promoter (TRAP-tdTomato mice) or the adenosine triphosphate (ATP)-driven proton pump (a3) promoter (a3-GFP mice) were employed [8, 11–13]. Mature osteoclasts express a large amount of TRAP; therefore, TRAP was used as an osteoclast marker for histological evaluation. Meanwhile, a3 is a subunit of vacuolar-type H^+^-ATPase (V-ATPase) on the osteoclast cell surface. V-ATPase forms acidic environments in the bone resorption cavity by transporting H^+^. To visualize osteoclast precursors, knock-in mice (CX_3_CR1-EGFP mice) expressing enhanced green fluorescent protein (EGFP) under the promoter of fractalkine receptors (CX_3_CR1) were used [6, 7, 14]. CX_3_CR1-positive cells include osteoclast precursors. In observing osteoclasts and osteoclast precursors in the bone marrow, bone matrix and collagen fibers were visualized by detecting a nonlinear optical process called second-harmonic generation [15, 16] using a two-photon microscopy [10, 13, 17–20].

**Fig. 2:**
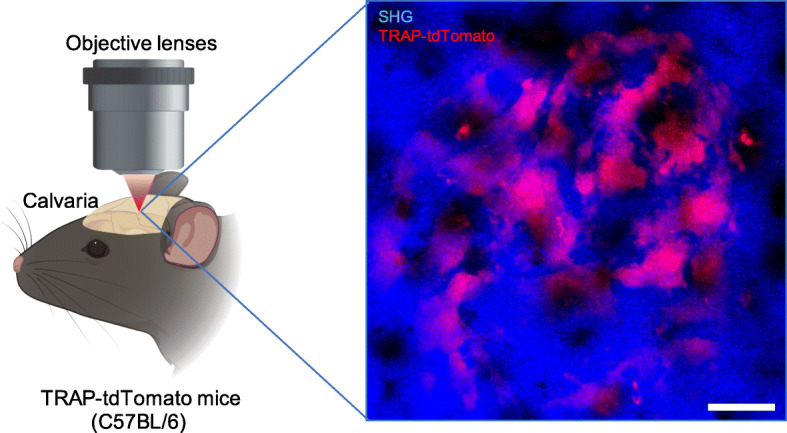
Observation of osteoclasts in the bone marrow. Osteoclasts were observed using an upright two-photon microscopy. This is a representative result. Blue: second-harmonic generation (SHG), Red: TRAP-tdTomato, Scale bar: 30 μm

To observe the behaviors of osteoclasts and osteoclast precursors, we performed and developed original intravital imaging techniques of a cranium with a thin bone matrix (Fig. 2) [6–9]. While observing the behaviors of osteoclasts and their precursors, we revealed that the migration of precursors was controlled by sphingosine-1-phosphate (S1P) in the blood [6, 7], and activated vitamin D significantly suppressed bone destruction by controlling their migration via an S1P mediator [9]. In addition, to observe the bone-resorbing ability of osteoclasts, we developed a pH-sensitive fluorescent probe [8, 11, 21, 22]. This probe fluoresces in response to acidic environments in the bone resorption cavity. We identified two distinct functional types of osteoclasts based on their bone-resorbing ability and motility [8]. Osteoclasts that strongly adhere to the bone surface and actively dissolve the bone matrix are bone-resorbing (R) cells. The other osteoclasts, which loosely adhere to the bone surface with high motility and have a low bone-resorbing ability, are non-resorbing (N) type cells. Intravital imaging with a pH-sensitive fluorescent probe has elucidated the conversion from N-type to R-type cells, which enhances osteoclastic bone destruction.

Intravital imaging is an effective technology for observing the in vivo pharmacological actions of drugs. We performed pharmacometrics of a wide variety of drugs and revealed the different functional mechanisms of each drug, not only in the steady-state but also under inflammatory conditions and in an experimental disease model [9, 23, 24]. Intravital imaging by using two-photon microscopy can reveal beneficial information about living cells from living mice.

### Imaging of pathological osteoclasts in inflamed synovium in subjects with arthritis

**Fig. 3:**
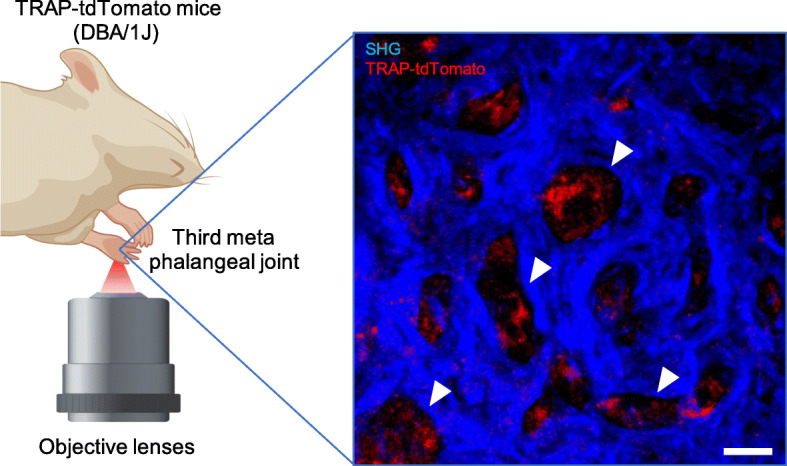
Observation of osteoclasts in the interface between the inflamed synovium and bone surface. Osteoclasts were observed using an inverted two-photon microscopy. This is a representative result. Blue, second-harmonic generation (SHG); Red, TRAP-tdTomato; Arrow heads, representative bone resorption bits; Scale bar: 30 μm

In arthritis, osteoclasts exhibit different behaviors compared to those under steady-state conditions. It is caused by an unusual environment such as surrounding immune cells and fibroblasts in the pannus microenvironment in the absence of osteoblasts. Observing the dynamics of osteoclasts at the pannus–bone interface is important for better understanding the pathogenesis of arthritis. Therefore, we observed the dynamics of osteoclasts in arthritis with two-photon microscopy and fluorescent transgenic mice [23]. The observation of osteoclasts in the arthritic joint is difficult because an excitation laser cannot reach the pannus–bone interface due to the long distance between the surface of the synovium and the pannus–bone interface, as well as the heterogenous and hypervascular nature of the inflamed synovium. To overcome this problem, we established a novel system for observing osteoclasts in the joints of arthritic mice [23]. First, we observed the third meta phalangeal joint of the forepaw. The synovium of this area is thin enough to permit penetration of an excitation laser from a two-photon microscopy. Next, we directly exposed the interface between the bone surface and inflamed synovium by cutting the skin and tendon of the middle digit using micro-scissors under a stereoscopic microscope. Using an inverted two-photon microscopy, we observed the bare area, where the bone was exposed to the synovium without the cartilage covering and therefore was vulnerable to erosion. We succeeded in visualizing small bone resorption pits (diameter = 20–50 μm) caused by the pathological bone destruction of osteoclasts (Fig. 3) and observed the bone resorption activity of osteoclasts in the pits of the bone surface using a pH-sensitive fluorescent probe. After subcutaneous administration of the probe, we observed fluorescence from the acidic area in these pits [23]. These results showed that the features of pathological osteoclasts at the pannus–bone interface under arthritic conditions are distinctive from those in the bone marrow under steady-state conditions, which interact and migrate with osteoblasts.

### Identification of novel osteoclast precursors: AtoMs

The concept that osteoclasts under arthritic conditions are the same as those under physiological conditions in the bone marrow has been widely accepted. However, the results of intravital imaging studies indicate that the resultant osteoclasts are more heterogeneous than presently thought, as are their differentiation pathways. There are CX_3_CR1-positive macrophages in the synovial lining [25]; however, a considerable number of inflammatory monocytoid cells that ingress into the synovium under arthritic conditions also express CX_3_CR1 [10]. It is unknown which CX_3_CR1-positive population differentiates into osteoclasts and causes bone destruction in arthritis. Therefore, we investigated the details of osteoclasts and their precursors in an arthritis model [10]. First, we used the collagen-induced arthritis (CIA) model and backcrossed the CX_3_CR1-EGFP/TRAP-tdTomato transgenic mice onto the DBA/1J background to identify the osteoclast precursor population in the inflamed synovium. Because the synovium is a tiny tissue surrounded by heterogenous organs, such as the fat tissue and bone marrow cells of the patella and femur, it is difficult to purely isolate the inflamed synovium from the bare area without contaminating other tissues. To overcome this problem, we developed an original protocol to expose the inflamed synovium on the knee joints by removing the patella, patellar ligaments, and quadriceps femoris muscles using microscissors and a stereoscopic microscope. After the onset of arthritis, we isolated synovial tissues at the bare area from the inflamed synovium with these methods. Using the ex vivo culture system of the isolated inflamed synovium, we directly visualized CX_3_CR1-positive cells differentiating into mature osteoclasts by gradually expressing TRAP-tdTomato and subsequently fusing with other TRAP-positive cells. Next, we investigated the origin of CX_3_CR1-positive cells using bone marrow chimeric mice and a parabiotic model between CX_3_CR1-EGFP/TRAP-tdTomato double-transgenic and wild-type mice. In chimeric mice, osteoclasts and precursors in inflamed joints were derived from bone marrow cells. In the parabiotic model, CX_3_CR1-positive precursors and mature TRAP-tdTomato-positive osteoclasts were detected in the inflamed joints of wild-type mice. These results suggest that CX_3_CR1-positive cells are derived from bone marrow cells and differentiate into mature osteoclasts after migrating to the inflamed synovium via blood circulation.

**Fig. 4:**
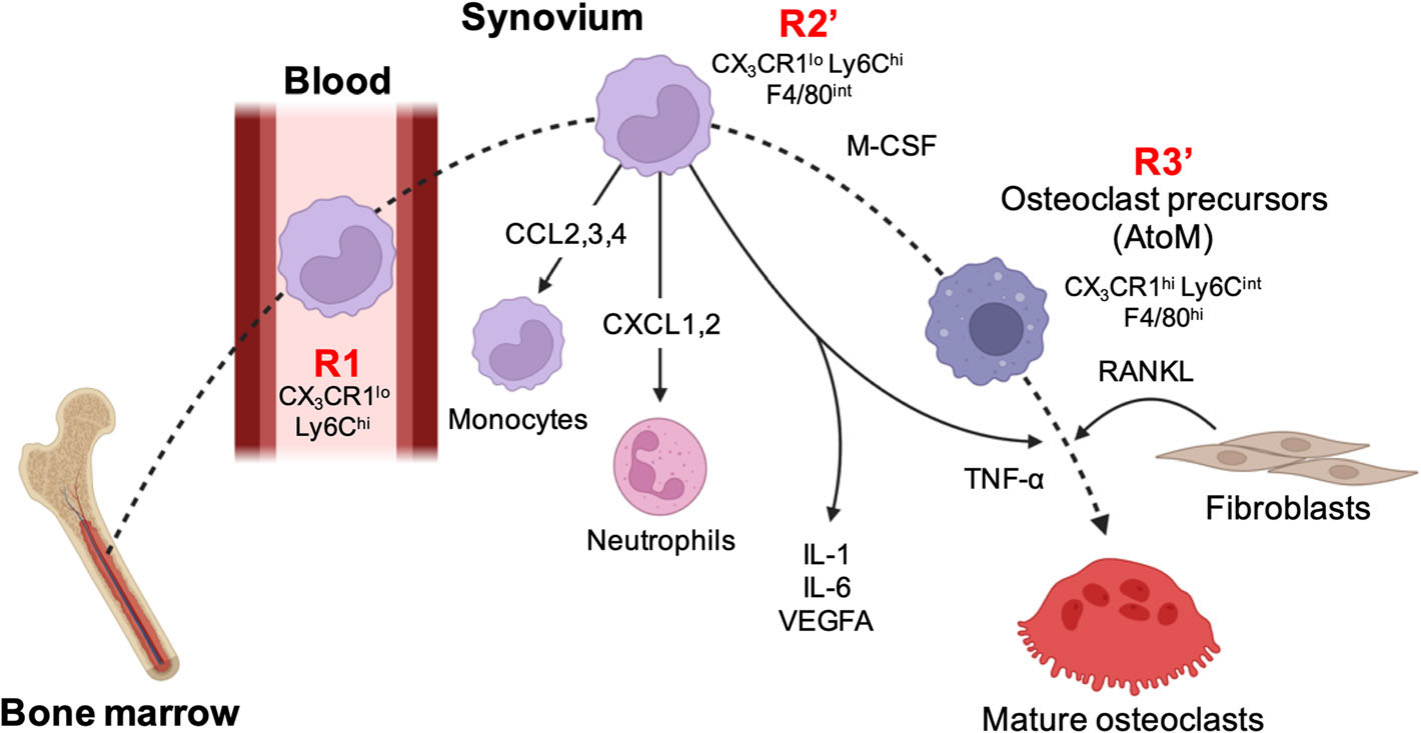
Newly identified osteoclast precursors in inflamed synovium differentiate into pathological osteoclasts. Schematic of the differentiation pathway of pathological osteoclasts and AtoMs in arthritis

To analyze CX_3_CR1-positive osteoclast precursors in more detail, we performed flow cytometry analysis of CX_3_CR1-positive cells in the blood, bone marrow, and inflamed synovium of CIA mice. The levels of CX_3_CR1^lo^ Ly6C^hi^ cells in the blood and bone marrow, known as inflammatory monocytes [26], were significantly increased. Moreover, in the inflamed synovium, CX_3_CR1^hi^ Ly6C^int^ cells and CX_3_CR1^lo^ Ly6C^hi^ cells were detected. Together, these results suggest that CX_3_CR1^lo^ Ly6C^hi^ cells (R1) in the blood migrate to the inflamed synovium (R2) and differentiate into CX_3_CR1^hi^ Ly6C^int^ cells (R3) in the inflammatory microenvironment (Fig. 4). Furthermore, because some cells are in a transition state between R2 and R3, we redefined R2’ cells and R3’ cells based on the expression level of F4/80. R3’ cells expressed cell surface markers for both macrophages and dendritic cells such as F4/80, I-A/I-E, CD80/86, and CD11c. The osteoclastogenic ability of R1, R2,’ and R3’ cells were analyzed by directly stimulating them with RANKL in vitro, which showed that R3’ cells have prominent osteoclastogenic ability among these cell subsets. Notably, combined stimulation with RANKL and TNF-α was most efficient at inducing the osteoclastogenesis of R3’ cells. These results suggest that pathological osteoclast precursors are included in the R3’ cells of the inflamed synovium; we named the R3 cells AtoMs [10]. Because AtoMs express cell surface markers related to antigen presentation in contrast to the physiological osteoclast precursors in the bone marrow, AtoMs may share functional characteristics with osteoclast precursors and dendritic cells, thereby promoting inflammation by presenting auto-antigens to further exacerbate osteoclastogenesis.

### Single-cell RNA sequencing analysis of AtoMs

Single-cell RNA sequencing analysis (scRNA-seq) is a useful tool that can predict the cell genealogy for an objective cell population [27]. However, it has never been used on osteoclast precursor cells in situ and the precise number of cells differentiating into mature osteoclasts in vivo among these precursor populations remains unknown. Therefore, we isolated AtoMs and performed the scRNA-seq analysis, which identified a specific subpopulation that highly expresses osteoclast marker genes, such as *Acp5*, *Atp6v0d2*, *Mmp9*, *Itgb3*, and *Ctsk*; this subpopulation constituted about 10% of AtoMs (approximately 1000 cells per mouse) in the inflamed synovium [10]. Furthermore, this subpopulation of AtoMs selectively express forkhead box M1 (Foxm1), which plays important roles in the cell invasion and carcinogenesis of a wide range of cancers [28], and upstream regulator analyses of the bulk-RNA seq data of AtoMs also showed that this transcriptional factor has a regulatory role in AtoMs. In the arthritis model of *Foxm1* global knock-out mice (*Foxm1*^flx/flx^*Rosa26*^CreERT2^), articular bone erosion was partially alleviated and the joint swelling was alleviated. In addition, the FoxM1 inhibitor thiostrepton [29–31] significantly suppressed bone destruction in arthritis and differentiation into osteoclasts. Notably, thiostrepton did not affect physiological osteoclasts and bone remodeling occurred as normal. These results suggest the involvement of *Foxm1* in arthritic bone destruction, although it has yet to be revealed whether it has a direct role in an osteoclastogenesis or indirect role by suppressing inflammation.

### Future perspective of AtoMs

Intravital imaging has a high potential to elucidate the cell dynamics and relationship of other cells or environments. However, current imaging techniques for observing inflamed synovium have limitations such as a difficult and time-consuming procedures and unstable visual fields. It is essential to overcome these problems to further reveal the migration patterns and differentiation of AtoMs into pathological osteoclasts in situ. AtoMs have unique signatures as osteoclast precursor cells in the joint that are distinctive from those in the bone marrow. We are currently analyzing the more detailed regulatory mechanisms of AtoMs. Although thiostrepton significantly suppressed osteoclastogenesis by AtoMs, the suppressive efficacy of FoxM1 expression is still partial in AtoMs and it is important to develop novel drugs with higher potency to become an effective treatment for RA, which is expected to specifically suppress pathological osteoclastogenesis in arthritis.

## Conclusion

Osteoclasts are tightly attached to the bone surface and are difficult to collect and analyze from living animals. Particularly under inflammatory conditions, their populations, pathways, and functions may greatly differ. It is very difficult to reproduce the pathological synovial microenvironment in vitro. Intravital imaging would be a powerful tool for identifying novel cells and understanding the pathology of inflammatory disease in vivo.

## Data Availability

The data of this study are available from the corresponding author on request.
